# The metamorphic transition of the frog mouth: from tadpole keratinized mouthparts to adult teeth

**DOI:** 10.1098/rsos.251196

**Published:** 2025-09-03

**Authors:** Daniel J. Paluh, Madeline Brinkman, Kyliah Gilliam-Beale, Daniela Salcedo-Recio, Jacob Szafranski, James Hanken, Gareth J. Fraser

**Affiliations:** ^1^ Department of Biology, University of Dayton, Dayton, OH, USA; ^2^ Department of Biology, University of Florida, Gainesville, FL, USA; ^3^ Museum of Comparative Zoology, Harvard University, Cambridge, MA, USA

**Keywords:** Anura, odontogenesis, development, novelty, Dollo's law, evo-devo

## Abstract

Teeth have been a prominent feature of most vertebrates for 400 million years, and the core regulatory network underlying embryonic tooth formation is deeply conserved. In frogs, however, odontogenesis is delayed, occurring instead during the postembryonic metamorphosis and resulting in teeth that are restricted to the upper jaw and palate. Developmental-genetic mechanisms that underlie tooth formation in frogs are poorly understood. We assessed if the genes underlying odontogenic competence are conserved in the late-forming teeth of frogs; if unique keratinized mouthparts, which function as an alternative feeding tool in anuran larvae, impede tooth induction; and if transient tooth rudiments form in the anuran mandible. We demonstrate that the induction of tooth development is conserved in the frog upper jaw, which displays odontogenic band expression patterns comparable to those of other vertebrates. There is, however, no evidence of tooth development initiating in the mandible. Adult teeth emerge before larval mouthparts degenerate, but their location may be spatially constrained by keratin. Gene expression patterns of keratinized mouthparts and teeth overlap. We hypothesize that the novel mouthparts of tadpoles, which we characterize as ectodermal appendages, may have originated by partially co-opting the developmental program that typically mediates development of true teeth.

## 1. Introduction

Teeth are complex, mineralized structures composed of a pulp cavity, dentin and enamel. They originated in stem gnathostomes over 400 million years ago [1]. The developmental genetics of tooth formation has been studied extensively in chondrichthyans, teleosts and amniotes, and the highly complex core gene regulatory network (GRN) underlying odontogenesis is deeply conserved [2–5]. In fishes and amniotes, this programme is regulated by several signalling pathways, including Hedgehog (Hh), Wingless (Wnt), Bone morphogenic (Bmp), Fibroblast growth factor (Fgf) and Ectodysplasin (Eda) [2–5]. Tooth development in the embryonic jaws of fishes, salamanders and most amniotes begins with the formation of an odontogenic band (OB), a precursor molecular expression pattern marked by the genes *Shh*, *Pitx2* and *Sox2* which defines the region of oral epithelium that is competent to form teeth [2,4–8]. The OB later forms the dental lamina [9], a band of thickened oral epithelium that gives rise to tooth buds. The core regulatory network underlying the induction, differentiation and morphogenesis of teeth is highly conserved within the oropharyngeal cavity (e.g. upper versus lower jaw, oral versus palatal teeth) [3,8].

Because teeth play a crucial role in the acquisition and processing of food, they are broadly conserved among chondrichthyans, actinopterygians and sarcopterygians, although their shape, size, location and number vary widely owing to broad variation in feeding mechanisms. Complete loss of teeth is rare in most vertebrate clades, having evolved only three times in living actinopterygian fishes and five times in living amniotes [10]. However, complete edentulism has occurred independently in more than 20 lineages of living frogs [10]. Partial tooth loss—when functional teeth fail to develop in a specific region of the oropharyngeal cavity—is more common, including loss of oral teeth in cyprinid fishes [11], loss of canines and most premolars in rodents [12] and loss of lower jaw teeth in frogs [10]. Compared with the rich knowledge of odontogenesis in most vertebrate clades, the development and genetics of tooth formation in frogs are poorly understood. Additionally, the mechanisms underlying instances of complete and partial tooth loss in frogs have not been explored.

Among living amphibians, all salamanders and caecilians possess teeth on both upper and lower jaws [10]. Frogs, however, are distinct, for while they variably possess teeth on the maxillary and premaxillary bones of the upper jaw and on the vomerine bone of the palate, all species but one lack lower jaw teeth [10]. The lone exception is *Gastrotheca guentheri*, in which mandibular teeth re-evolved [13,14]. Frog teeth form after hatching, during metamorphosis [15], unlike other vertebrates in which tooth development occurs in the embryo and coincides with primary mouth formation [8,16]. The delayed timing of tooth development in frogs decouples odontogenesis from the formation of the primary mouth. Dissociation of these two developmental processes could be consequential because many of the same genes are thought to regulate both mouth and dental developments [17]. Upper jaw tooth induction occurs in late larval stages in two species of clawed frogs, *Xenopus laevis* [18] and *Xenopus tropicalis* [17]. However, the timing of tooth initiation has not been precisely identified in any other frog species and is generally stated to occur during metamorphosis [15], which is a complex and dynamic phase of development that typically spans several weeks or longer. An analysis of the developmental genetics of tooth induction in *X. tropicalis,* the only published study of this topic in anurans, failed to document an OB marked by *Shh* expression in the upper jaw [17], suggesting that the otherwise conserved genetic mechanism underlying odontogenic competence may be modified in frogs.

Delayed tooth development in frogs is a likely consequence of the unique feeding apparatus of tadpoles, and especially its keratinized mouthparts, because a non-keratinized oral epithelium is thought to be required for initiation of odontogenesis [19,20]. The feeding apparatus is considered an evolutionary novelty and key innovation [21] that enables frog larvae to feed on plant material and detritus. In most tadpoles, the mouth is surrounded by keratinized jaw sheaths (i.e. horny beak), several rows of keratodonts (i.e. keratinized labial ‘teeth’) and sensory papillae on the upper and lower labia of the oral disc. The jaw sheaths and keratodonts are composed of several epidermal cell types stacked into a column [22–24]. The basal cells are proliferative, enabling continuous replacement of these structures [25,26]. The evolutionary origin of the keratinized mouthparts is entirely unknown [27], as are the developmental genetics that mediate their formation. *Xenopus*, a popular anuran model in molecular and developmental biology, lacks these keratinized structures, which have impeded their study.

**Figure 1.**
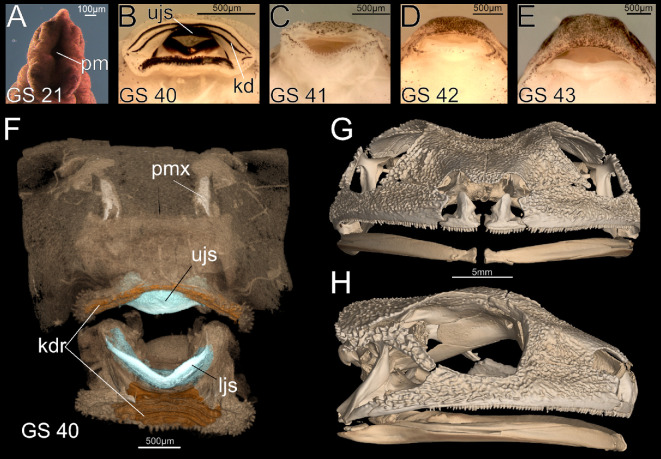
Anatomy and development of the feeding apparatus in the Cuban tree frog, *Osteopilus septentrionalis*. (A–E) Progressive development of the mouth: stomodeum (primary embryonic mouth; A), tadpole oral disc with keratinized mouthparts (B), atrophy of keratinized mouthparts (C), and formation of post-metamorphic frog jaws (D, E). (F–H) Micro-computed tomography scans of tadpole feeding apparatus (F) and adult skull with a toothed upper jaw and edentate mandible (G and H). Abbreviations: kd, keratodonts; kdr, keratodont ridges; ljs, lower jaw sheath; pm, primary mouth; pmx, premaxilla; ujs, upper jaw sheath. See the electronic supplementary material, Methods for scanning protocol.

In this study, we characterize the development of first-generation teeth in the Cuban tree frog, *Osteopilus septentrionalis*. This metamorphosing anuran has a typical tadpole with keratinized mouthparts and an adult with teeth on the upper jaw but a toothless mandible (figure 1). We evaluate both histological anatomy and expression of several key genes across a comprehensive developmental series to (i) determine the precise timing of odontogenesis in the upper jaw; (ii) assess if an OB, marked by the genes *Shh*, *Pitx2* and *Sox2* and comparable to that in other toothed vertebrates, forms on the upper jaw; (iii) seek evidence of inductive or inhibitory interactions between the keratinized mouthparts of tadpoles and developing teeth; and (iv) evaluate if tooth development is initiated on the lower jaw, coincident with upper jaw tooth formation, but is then abandoned.

## 2. Results

We investigated tooth development in the Cuban tree frog, *O. septentrionalis*. Adults of this species have a typical anuran dentition characterized by bicuspid, pedicellate teeth on paired premaxillae, maxillae and vomers but an edentate lower jaw (figure 1; [28]). Tadpoles of this species have a typical oral disc, which includes keratinized upper and lower jaw sheaths and keratodonts above and below the jaws. We staged all specimens following the Gosner system (Gosner stage [GS]; [29]), which is widely used to characterize embryonic (GS 1−22), larval (GS 23−40) and metamorphic (GS 41−46) stages of metamorphosing anurans (excluding the model taxon *Xenopus*). An ontogenetic series was obtained, ranging from the final phases of tadpole development (GS 40) to fully metamorphosed froglets (GS 46). A total of 45 specimens were examined histologically to evaluate the developmental transition of the anuran mouth during metamorphosis.

### 2.1. Establishing odontogenic competence on the upper jaw

**Figure 2.**
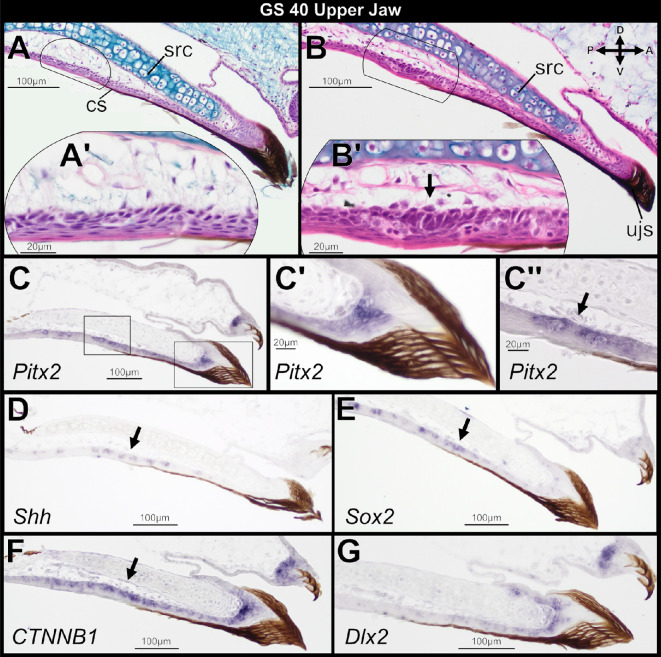
Sagittal sections through the larval upper jaw of two *Osteopilus septentrionalis,* Gosner stage (GS) 40, stained with haematoxylin and eosin (H&E) plus Alcian blue (A, B) and *in situ* hybridization (C–G). The earliest histological indication of tooth induction occurs with the formation of a localized epithelial thickening (arrow in B’). *Pitx2*, *Shh*, *Sox2* and *CTNNB1* mark the zone of upper jaw oral epithelium where tooth buds will form (arrows in C“, D, E, and F). *Pitx2*, *CTNNB1* and *Dlx2* are also highly expressed in the tadpole jaw sheath and keratodonts (C’, F and G). Insets (A’, B’, C’ and C’’) show higher magnification detail of regions outlined in (A), (B) and (C). Abbreviations: cs, cornified sheath cells; src, suprarostral cartilage. Orientation axes in (B): D, dorsal; V, ventral; A, anterior; P, posterior.

In *O. septentrionalis*, GS 40 can be considered the final ‘tadpole’ stage; the larval feeding apparatus, keratinized mouthparts and vent tube are still present, but hindfoot tubercles are beginning to form (figure 1B,F). We evaluated the oral histology of the upper jaw in seven GS 40 tadpoles and documented the beginning of tooth development in several of them. Four specimens show no evidence of tooth development, but the remaining three show the earliest histological indication of tooth development: formation of a localized thickening in the oral epithelium and condensation of underlying mesenchyme (figure 2A,B). The epithelial thickening will later form the dental lamina, and the condensing mesenchyme will give rise to the dental papilla.

Prior to tooth induction, the mucosa of the oral cavity roof adjacent to the mouth is composed of stratified squamous epithelium with a cornified outermost layer that is contiguous with the keratinized upper jaw sheath of the tadpole feeding apparatus (figure 2A, black cornified layers). Loose, uncondensed mesenchyme separates the oral epithelium from the suprarostral cartilage of the upper jaw. Following induction of tooth development, cells of the basal layer of the oral epithelium have transitioned to a columnar shape, producing a localized epithelial thickening at the posterior extent of the keratinized jaw sheath cells (figure 2B, arrow). The underlying mesenchyme is also beginning to condense, and abundant dividing cells (i.e. mitotic figures) within the oral epithelium suggest high cell proliferation rates.

Expression of several genes known to mediate tooth initiation accompanies the above histological changes. *Pitx2* is the earliest epithelial marker of tooth competence known in tetrapods [30]. At GS 40, the gene is expressed in the upper jaw oral epithelium prior to any histological indication of tooth development (figure 2C,C’’). Unexpectedly, *Pitx2* is also highly expressed within epithelial basal cells that support the upper jaw sheath and keratodonts of the keratinized feeding apparatus (figure 2C,C’). This expression is asymmetric—lingually in the jaw sheath and labially in the keratodonts. *Pitx2* expression is continuous between the epithelial base of the upper jaw sheath and the region where tooth buds will form. *Shh* is weakly expressed in the upper jaw oral epithelium before tooth development but is absent in the epithelial cells that support the keratinized jaw sheath and keratodonts (figure 2D). *Sox2* is expressed in the oral epithelium preceding tooth development and is also weakly expressed in the epithelial cells at the base of the keratodonts (figure 2E). *CTNNB1*, which encodes for β-catenin protein, is strongly expressed in the upper jaw oral epithelium, the upper jaw sheath and keratodonts (figure 2F). Unlike *Pitx2*, *CTNNB1* is more widely expressed throughout the basal cells of the jaw sheath and keratodonts, as well as underlying mesenchyme. Finally, *Dlx2* is expressed within the epithelial basal cells and underlying mesenchyme of the keratinized jaw sheath and keratodonts (figure 2G). *Dlx2* expression within the oral epithelium is limited to the anterior region that underlies the jaw sheath; unlike *Pitx2* and *CTNNB1*, it does not extend posteriorly to where the localized epithelial thickenings will form.

### 2.2. Emergence of the dental lamina and first-generation teeth on the upper jaw

**Figure 3.**
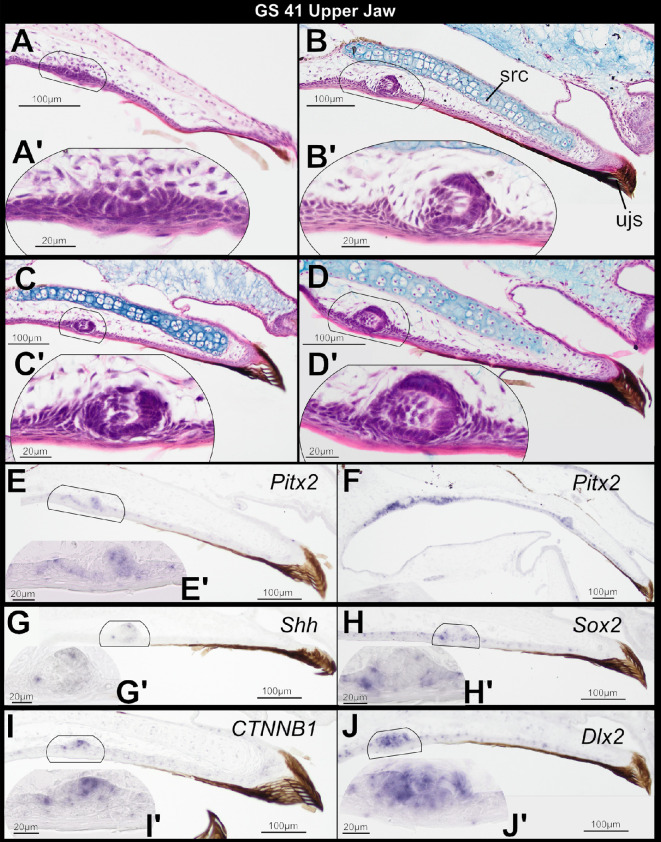
Sagittal sections through the larval upper jaw of four *Osteopilus septentrionalis* specimens*,* Gosner stage (GS) 41, stained with H&E plus Alcian blue (A–D) and *in situ* hybridization (E–J). The dental lamina emerges (A) and tooth placodes form (B–D) coincident with keratinized mouthparts. *Pitx2*, *Shh*, *Sox2*, *CTNNB1* and *Dlx2* are expressed in emerging tooth placodes (E–J). Insets (A’–J’) show higher magnification detail of regions outlined in (A–J).

GS 41 is characterized by atrophy of the keratinized mouthparts and vent tube (figure 1C). Of the nine GS 41 specimens sectioned, all of which lack a vent tube, keratinized mouthparts are retained in six but undergoing atrophy in three. Odontogenesis has begun in the upper jaw of all specimens. Tooth buds first appear in parasagittal sections ventral to distal and midshaft regions of the suprarostral cartilages; there is no indication of tooth development in midline sections where these paired cartilages meet. The earliest stage of dental lamina emergence and tooth placode formation is visible in some specimens: labial and lingual edges of the columnar basal cells that delimit the oral epithelial thickening are invaginating into the underlying ectomesenchyme, where they form a concave plate (figure 3; described as an inverted trough in *Xenopus* [18]). The ectomesenchyme is condensing and protrudes apically into the centre of the thickened epithelium. In other specimens, the dental lamina has become asymmetric; the labial edge of the epithelial plate has invaginated more deeply and in a posterior direction, forming a roof and differentiating into the dental epithelium that encloses condensed mesenchymal cells that will form the dental papilla (figure 3B,C). These first-generation tooth buds develop superficially, near the surface of the oral mucosa, similar to dental lamina emergence in *Xenopus* [31]. The bicuspid crown pattern has emerged in tooth buds of more advanced specimens that retain the jaw sheath (figure 3D). The jaws undergo remodelling as the tadpole mouthparts degenerate, but development of first-generation tooth buds does not advance dramatically during this time. Coincident with the above histological changes, the genes *Pitx2*, *Shh*, Sox2, *CTNNB1* and *Dlx2* are expressed in the emerging dental lamina of the upper jaw (figure 3E–J). Expression of *Pitx2*, *Sox2*, *CTNNB1* and *Dlx2* is no longer detectable in epithelial cells that support the keratinized jaw sheath, which is beginning to degenerate. *Pitx2* is widely expressed within oral epithelium on the roof of the mouth, in effect connecting the emerging dental lamina that will give rise to tooth buds on the palate (vomerine teeth) and the dental lamina that will give rise to teeth on the upper jaw (figure 3F). *Dlx2* is expressed in epithelial cells delimiting the dental lamina, as well as in condensed mesenchyme that will differentiate into the dental papilla (figure 3J).

**Figure 4.**
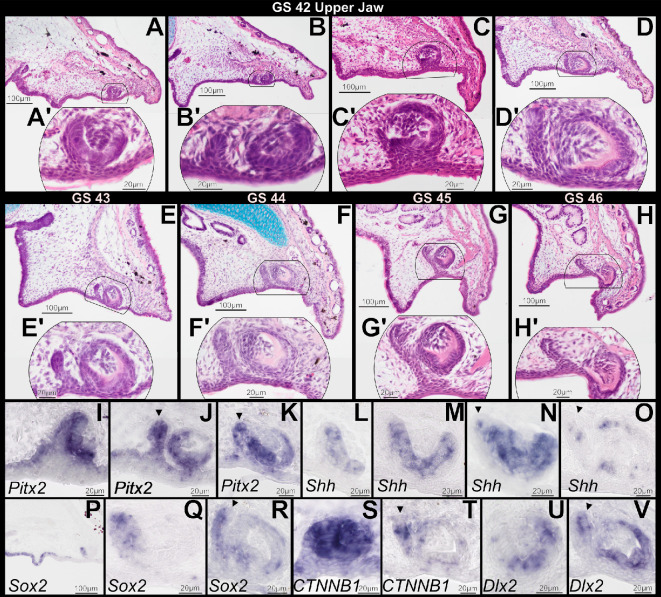
Sagittal sections through the upper jaw of *Osteopilus septentrionalis* during and immediately following metamorphosis, Gosner stages (GS) 42−46, stained with H&E plus Alcian blue (A–H) and *in situ* hybridization (I–V). Tooth buds in four GS 42 specimens at different stages of dental development (A–D). The successional dental lamina has emerged (B–D). The first signs of tooth matrix secretion by odontoblasts are also visible (C*,* D). Developing tooth buds do not become functional until after the completion of metamorphosis (E–H). *Pitx2*, *Shh*, *Sox2*, *CTNNB1* and *Dlx2* are expressed in dental tissues throughout morphogenesis (I–V). Arrowheads denote expression in the successional dental lamina. Insets (A’–H’) show higher magnification detail of regions outlined in (A–H).

GS 42 is characterized by emergence of fully formed forelimbs. The simple and narrow mouth still opens anterior to the external nares (figure 1D), and there are remnants of the tadpole oral disc. Teeth that collectively span several stages of development are visible along the upper jaw. Younger tooth buds are located nearer the midline, but all tooth buds at GS 42 have reached or surpassed the cap stage of morphogenesis (figure 4A,B), and the epithelial plate invaginates more deeply into the ectomesenchyme. A prominent successional dental lamina, which will give rise to future tooth generations, has emerged lingual to the first-generation teeth and connects the row of emerging tooth buds (figure 4B; electronic supplementary material, figure S1). In more advanced tooth buds, the dental epithelium has differentiated into inner and outer layers (figure 4C,D), and condensed ectomesenchymal cells have formed the dental papilla. Cells of the inner dental epithelium have differentiated into ameloblasts and dental papilla cells have differentiated into odontoblasts. The first indications of tooth matrix secretion by odontoblasts are visible (figure 4C,D).

At later metamorphic stages (GS 43−46), the jaws rapidly extend posteriorly (figure 1E), bringing the jaw joint posterior to the eye, while first-generation tooth buds continue dentinogenesis. The developing teeth migrate towards the ossifying maxillary and premaxillary bones, but at none of these stages have teeth implanted into bone or erupted through the oral mucosa (figure 4E–H). The successional dental lamina continues to invaginate more deeply into underlying ectomesenchyme and assumes a cup shape at its extremity (figure 4G). At the same time, condensing ectomesenchyme surrounds the extremity of the successional dental lamina (figure 4G,H), indicating the beginning of differentiation of second-generation tooth buds before metamorphosis is complete. First-generation teeth in frogs are non-pedicellate [32], and we saw no indication of pedicel formation.

During GS 42−46, the genes *Pitx2*, *Shh, Sox2*, *CTNNB1* and *Dlx2* are expressed in developing first-generation teeth (figure 4I–V). *Pitx2* is expressed in the inner and outer dental epithelia of tooth buds, as well as in the emerging successional dental lamina (figure 4I–K). *Shh* expression is seen within the inner dental epithelium of tooth buds at the cap stage (figure 4M,N) and marks the free, distal end of the successional dental lamina (figure 4N,O). At GS 42, *Sox2* is strongly expressed in olfactory and upper jaw oral epithelium posterior to the tooth buds, as well as in the lingual portion of the dental epithelium (figure 4P,Q). *Sox2* is also expressed in the emerging successional dental lamina (figure 4R). *CTNNB1* is strongly expressed in the dental epithelium and dental papilla during the cap stage (figure 4S). It is also present in the successional dental lamina and condensing ectomesenchyme at later stages (figure 4T). At GS 42, *Dlx2* expression is limited to the labial portion of the dental epithelium, but it later expands to the entire dental epithelium as well as the free, distolabial end of the successional dental lamina (figure 4U,V).

### 2.3. Evaluating odontogenic potential on the anuran lower jaw

We examined the lower jaw in all 45 sectioned specimens for any histological evidence of odontogenesis coincident with tooth formation in the upper jaw. The tadpole mandible has a complex shape and is segmented into two parts: anteromedially, a V-shaped infrarostral cartilage supports the keratinized lower jaw sheath, while posteriorly and laterally, a pair of transversely oriented Meckel’s cartilages articulate with the infrarostral via an intramandibular commissure [21]. During metamorphosis, infrarostral and Meckel’s cartilages fuse to form a single rod of lower jaw cartilage.

**Figure 5.**
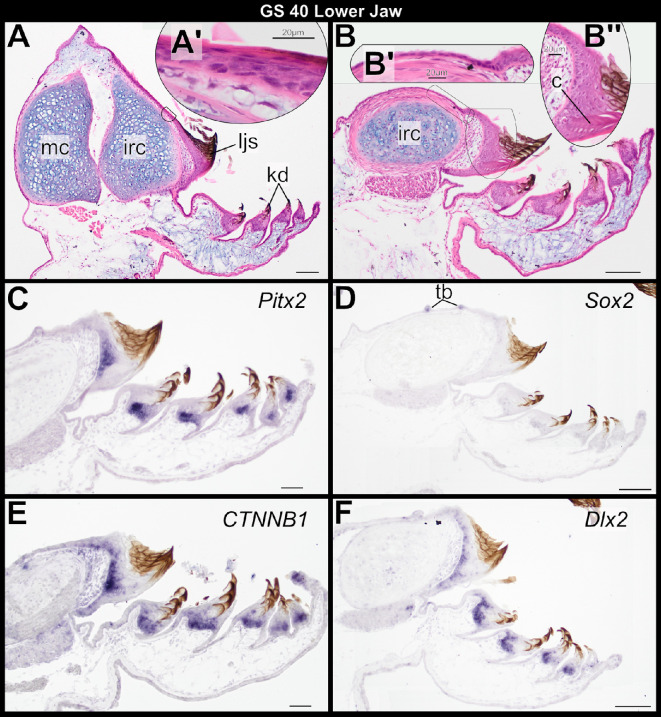
Sagittal sections through the larval lower jaw of *Osteopilus septentrionalis*, Gosner stage (GS) 40, stained with H&E plus Alcian blue (A, B) and *in situ* hybridization (C–F). Insets (A’) and (B’) show higher magnification detail of the lower jaw oral epithelium overlying the infrarostral cartilage; (B”) shows detail of the basal cells, sheath cells and cone cells that form the jaw sheath. *Pitx2*, *CTNNB1* and *Dlx2* are highly expressed in the tadpole jaw sheath and keratodonts (C, E and F). *Sox2* is expressed in the larval taste buds overlying the infrarostral cartilage and in keratodont progenitor cells. Abbreviations: c, cone cells; irc, infrarostral cartilage; mc, Meckel’s cartilage; tb, taste buds. Scale bar, 100 µm.

Parasagittal sections through articulating Meckel’s and infrarostral cartilages at GS 40 reveal a simple, squamous epithelium overlying the lingual side of Meckel’s cartilage (figure 5A). In this same plane, a stratified, squamous epithelium that is contiguous with the lower jaw sheath overlies the labial side of the infrarostral cartilage (figure 5B). Histologically, this epithelium resembles the oral epithelium of the upper jaw before odontogenesis (figure 2A). In sections closer to the midline, most of the oral epithelium overlying the infrarostral cartilage is simple and squamous (figure 5). By contrast, epithelia associated with the keratinized lower jaw sheath are complex, including columnar basal keratinocytes, several layers of stratified sheath cells that form the lingual and labial surfaces of the jaw sheath and a central column of proliferating cone cells, which form the cutting edge of the jaw (figure 5C). None of the GS 40 specimens shows any histological evidence of a localized epithelial thickening overlying Meckel’s or infrarostral cartilage, nor is there any other indication of odontogenesis in the lower jaw. However, gene expression patterns within the keratinized mouthparts of the lower jaw are comparable to those in the upper jaw at this stage. *Pitx2*, *CTNNB1* and *Dlx2* are highly expressed in the lower jaw sheath and keratodonts (figure 5C,E,F). *Pitx2* expression is asymmetric—lingually in the jaw sheath but labially in the keratodonts—while *CTNNB1* and *Dlx2* expression is more widespread in the basal and suprabasal epithelial cells that support these structures. Expression of these markers is limited to epithelial cells associated with the keratinized lower jaw sheath and does not extend posteriorly to the oral epithelium that overlies the infrastrostral cartilage, a pattern that differs markedly from *Pitx2* and *CTNNB1* patterns in the upper jaw. *Sox2* is weakly expressed in basal cells underlying the keratodonts and is seemingly absent from the lower jaw sheath (figure 5D), but papillae in the oral epithelium and overlying the infrarostral cartilage are positive for *Sox2*. These papillae probably house taste buds [33], and *Sox2* is a well-known taste-bud marker in vertebrates [34]. No *Shh* expression was detected in lower jaw oral epithelium, lower jaw keratinized sheath or keratodonts.

**Figure 6.**
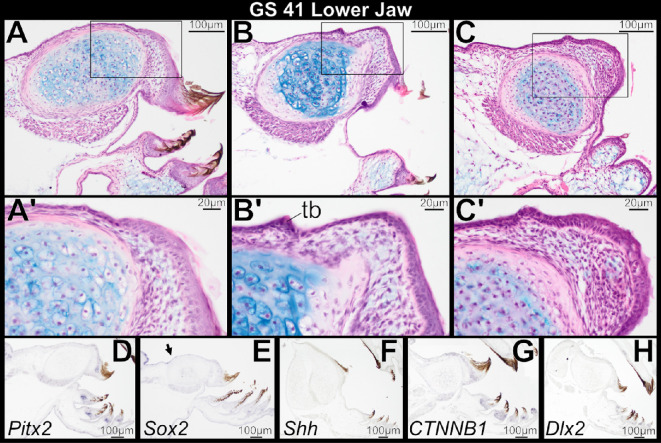
Sagittal sections through the larval lower jaw of *Osteopilus septentrionalis*, Gosner stage (GS) 41, stained with H&E plus Alcian blue (A–C) and *in situ* hybridization (D–H). Keratinized mouthparts degenerate (A, B), and columnar basal epithelial cells are a relic of the lower jaw sheath (C). Epithelium overlying the infrarostral cartilage transitions from single- to multi-layered. There is no histological evidence of mandibular tooth formation. *Pitx2*, *CTNNB1* and *Dlx2* are weakly expressed in basal cells of the keratinized jaw sheath and keratodonts (D, G and H). *Sox2* expression expands anteriorly from the tongue epithelium towards the oral epithelium (E, arrow). Insets (A’–C’) show higher magnification detail of regions outlined in (A–C).

**Figure 7.**
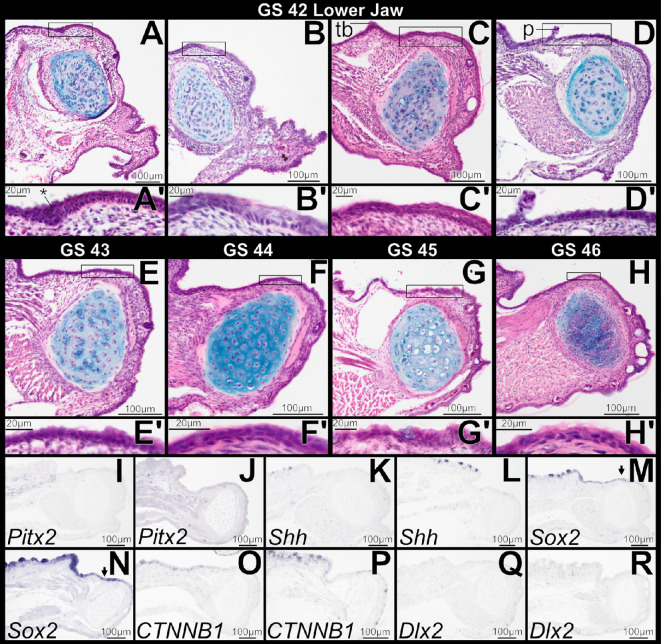
Sagittal sections through the lower jaw in *Osteopilus septentrionalis* during and immediately following metamorphosis, Gosner stages (GS) 42−46, stained with H&E plus Alcian blue (A–H) and *in situ* hybridization (I–R). Epithelium overlying lower jaw cartilage is multi-layered; there is no histological evidence of mandibular tooth formation. *Pitx2*, *Shh*, *CTNNB1* and *Dlx2* are absent from mandibular oral epithelium (excluding tongue taste discs; I–L, O–R). *Sox2* is expressed in oral epithelium of the lower jaw (M and N, arrows). Insets (A’–H’) show higher magnification detail of regions outlined in (A–H). Abbreviations: p, papilla; tb, taste bud; *, invaginated epithelial thickening.

Lower jaw histology of GS 41 specimens that retain keratinized mouthparts is very similar to that at GS 40, and there is no sign of tooth development (figure 6A). As the tadpole feeding apparatus atrophies during this stage, epithelium that previously supported the lower jaw sheath at the oral/aboral boundary retains columnar basal cells, which produce a thickened area (figure 6B,C). This thickening is broader than the localized epithelial thickenings that form in the upper jaw; it does not form a concave epithelial plate and persist into GS 42. We interpret it as a relic of the tadpole lower jaw sheath and unrelated to odontogenesis. Posteriorly, epithelium overlying the infrarostral cartilage transitions from single- to multi-layered as the tadpole feeding apparatus is lost (figure 6B,C), and oral epithelium of the lower jaw retains a stratified architecture for the remainder of metamorphosis (GS 42−46; figure 7A–H). One GS-42 specimen has an apparent epithelial thickening that invaginates slightly into the underlying mesenchyme (figure 7A). However, the basal cells of this thickening are not columnar, and the tissue architecture is inconsistent with formation of a dental lamina as seen in the upper jaw. We regard it as the remnant of a larval papilla or taste bud that is being resorbed, as other GS 41 and 42 specimens retain papillae in this general region that are in various stages of degeneration (figures 6B and 7C,D). There is no histological evidence of mandibular tooth formation in any GS 42−46 specimen.

At GS 41, the genes *Pitx2*, *CTNNB1* and *Dlx2* are all weakly expressed in basal cells of the keratinized jaw sheath and keratodonts (figure 6D,G,H ). These markers, however, are not expressed in the oral epithelium posterior to the jaw sheath and overlying the infrarostral, and they are not seen in the lower jaw at GS 42, following degeneration of tadpole mouthparts (figure 7I,O,Q). Their absence persists through metamorphosis (figure 7J,P,R). *Shh* is not expressed in the lower jaw between GS 41 and 46 (figures 6F and 7K,L). *Sox2* expression expands anteriorly from the lingual epithelium towards the epithelium overlying the infrarostral cartilage beginning at GS 41 (figure 6E), and by GS 42 it is seen within the oral epithelium of the lower jaw (figure 7M). It persists within the oral epithelium to later stages (figure 7N). *Shh*, *Sox2* and *CTNNB1* are expressed in the developing taste buds of the tongue (figure 7L,N,P).

### 2.4. Formation of the primary mouth

**Figure 8.**
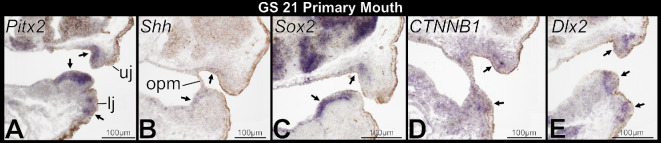
Sagittal sections through the primary mouth of embryonic *Osteopilus septentrionalis*, Gosner stage (GS) 21, following *in situ* hybridization (A–E). Arrows denote positive gene expression in the oral and aboral epithelium of upper and lower jaw primordia. Abbreviations: lj, lower jaw primordium; opm, oropharyngeal membrane; uj, upper jaw primordium.

Examination of GS 40−46 specimens revealed unexpected expression of several genes known to mediate tooth initiation in association with components of the keratinized feeding apparatus. We, therefore, assessed if expression of these same genes is associated with embryonic formation of the primary mouth (i.e. stomodeum) at GS 20−21 and preceding the development of keratinized jaw sheaths and keratodonts. These stages are characterized by rupture of the oropharyngeal membrane, which separates oral ectoderm from foregut endoderm [35,36], and by formation of the primordial mouth. *Pitx2*, *Shh*, *Sox2*, *CTNNB1* and *Dlx2* are all expressed within oral epithelium of the primordial upper and lower jaws (figure 8A–E). *Shh* and *Sox2* are limited to oral epithelium and foregut endoderm, whereas *Pitx2* and *Dlx2* have separate zones of expression in oral and aboral epithelium. *CTNNB1* is widely expressed in the epithelium and underlying mesenchyme of the primordial jaws.

## 3. Discussion

### 3.1. Dental competence and emergence of first-generation teeth of the frog upper jaw

Studies of turtles, birds and non-avian theropod dinosaurs suggest that the presence of keratinized tissue overlying the jaw epithelia may dictate the location of tooth formation or even entirely prevent it [19,37,38]. Our results with the Cuban tree frog support the hypothesis that keratin can dictate the location of tooth formation. While primary teeth begin to emerge on the upper jaw at GS 40 before the keratinized mouthparts degenerate, the presence of the upper jaw sheath appears to constrain the initial location of emerging tooth buds within the oral epithelium: the earliest epithelial thickenings and tooth placodes are positioned far from the oral/aboral boundary and posterior to the superficial, keratinized sheath cells that roof the anterior-most region of the oral mucosa (figures 2B and 3A–D). Following atrophy of the tadpole mouthparts and resorption of the suprarostral cartilage, additional tooth buds form closer to the tip of the upper jaw (figure 3).

In *X. laevis* and *X. tropicalis*, the initiation of tooth development occurs at Nieuwkoop and Faber (NF) stages 55 and 56, respectively [17,18]. Tooth formation then proceeds rapidly, with dental lamina emergence and the onset of dentinogenesis occurring at the same NF stage [18], which is comparable to our findings in *Osteopilus* at GS 41 (figure 3A–D). NF stages 55 and 56 are equivalent to GS 35−38 in *Osteopilus* and many other metamorphosing anurans [39], and they precede metamorphic climax by several weeks. Some authors suggest that onset of tooth formation in *Xenopus* is early compared with other frogs and attribute this to the lack of keratinized mouthparts in this lineage [40], but we regard this as unlikely. First, *O. septentrionalis* possesses tooth buds and jaw sheaths concurrently. Second, while Hertwig [41], in the earliest description of anuran teeth, reported tooth development in *Pelobates fuscus* beginning at GS 42, following the loss of keratinized mouthparts, the tooth buds he describes are already at the cap stage of morphogenesis, well after onset of tooth development. The only other histological studies of anuran odontogenesis examine replacement teeth that differentiate from the deeply invaginating successional dental lamina in adults [42,43]. More research is needed to assess variability in the timing of tooth induction across anuran diversity and determine if and how the formation of first-generation teeth is affected by keratinized tadpole mouthparts.

We tested for the expression of several key genes to assess if an OB precedes formation of teeth on the upper jaw in *O. septentrionalis*. The genes *Pitx2*, *Sox2* and *Shh* are expressed in oral epithelium at GS 40 (figure 2C–E), in the region where tooth placodes will form. Thus, frogs have dental precursor expression patterns consistent with an OB and comparable to those in chondrichthyans [4], teleosts [2,34], salamanders [8] and amniotes [6,12]. Our findings differ from those reported for *X. tropicalis*, in which *Shh* was not detected in upper jaw oral epithelium preceding tooth formation and was observed only in the inner dental epithelium of cap-stage tooth buds [17]. That study assessed gene expression by using whole-mount *in situ* hybridization, whereas we examined serial sections. In our preparations, *Shh* is weakly expressed in oral epithelium prior to tooth formation and during dental lamina emergence, and expression does not strengthen until the inner dental epithelium differentiates. Typically in vertebrates, *Pitx2*, *Shh* and *Sox2* are all strongly expressed in the oral epithelium and are easily detected as a horseshoe-shaped band of expression in the jaws of wholemount embryos [8,12,44,45]. Frogs may be unique in having a less pronounced OB. Alternatively, wholemount *in situ* hybridization may be less effective in post-embryonic specimens, which are larger and have less-transparent tissues. The gene expression patterns we report in *O. septentrionalis* reaffirm that the core gene regulatory network underlying tooth initiation is deeply conserved among vertebrates [4], including the late-forming teeth of anurans.

### 3.2. Is tooth development induced on the lower jaw?

We find no definitive histological or genetic evidence of tooth development initiating in the lower jaw of *O. septentrionalis* when teeth are forming on the upper jaw. We looked for expression of several key genes to assess if a transient OB forms on the mandible, but we did not detect such patterns in the oral epithelium overlying the lower jaw cartilages at any stage. *Sox2*, which regulates progenitor dental epithelial cells [6], is the only OB marker expressed within the mandibular oral epithelium, possibly indicating that the tooth GRN may not be completely suppressed in the lower jaw. In cichlids, *Sox2* dramatically expands in the lower jaw following chemical knockdown of Bmp and Shh signalling, which ultimately yields fewer teeth [34]. Thus, it is also possible that *Sox2* expression is expanded or dysregulated in the lower jaw of frogs owing to the absence of other molecular signals within the oral epithelium and the loss of dental competence.

The lack of tooth bud rudiments and absence of early odontogenic signalling in the lower jaw of frogs is unexpected because structures evolutionarily lost from adult vertebrates typically form at least partly during development [46,47]. Expression patterns consistent with a transient odontogenic band, and sometimes vestigial tooth rudiments, are well documented in vertebrates that have lost teeth, including those in which teeth are entirely absent (e.g. edentulous mammals [48], birds [49] and turtles [16,50]) and others in which tooth loss is spatially restricted (e.g. diastema teeth in rodents [12,51] and oral jaw teeth in cypriniform fishes [52,53]). For example, the tooth development programme can initiate at least partially in a subset of wild-type chick embryos through early odontogenic signalling in the oral epithelium, resulting in a vestigial dental lamina-like structure [49]. Mysticete whale embryos form tooth buds that can reach the bell stage of morphogenesis before degenerating [54]. Frogs may be unique in more fully suppressing the tooth development programme within the lower jaw because the formation of the primary mouth is decoupled from the onset of tooth development during metamorphosis. Embryonic expression of *Pitx2*, *Shh*, *Sox2*, *CTNNB1* and *Dlx2* in the primordial oral epithelium of both jaws during primary mouth development in *O. septentrionalis* may indicate that these genes are (i) additionally involved in regulating primary mouth formation, (ii) involved in the development of keratinized mouthparts, and/or (iii) involved in the establishment of epithelial competence to form teeth on both jaws at an early embryonic stage, but the programme never progresses far enough to form teeth in the mandible. Shh and β-catenin (*CTNNB1*) signalling are involved in primary mouth formation in embryonic *Xenopus* [55,56], but the role of these other markers in stomodeum development requires further investigation [36]. The genetics underlying keratinized mouthpart formation in frog embryos have not been studied previously, but these genes may be involved in inducing jaw sheath and keratodont development based on their expression in late-stage tadpoles. Further experimental work in diverse species is needed to evaluate the developmental genetics of the anuran mouth during primary mouth formation, the development of the tadpole feeding apparatus and the transition to post-metamorphic jaws to determine the interconnectivity and dynamics of these processes.

Of the nearly 8000 species of living frogs [57], only Guenther’s marsupial frog, *Gastrotheca guentheri*, possesses teeth on the dentary bone of the lower jaw. Mandibular teeth were lost in the common ancestor of frogs more than 200 million years ago [13], but they were later regained in *G. guentheri* or one of its direct ancestors during the Miocene epoch [13,14]. The presence of mandibular teeth in this species is a notable exception to Dollo’s law of irreversibility, which holds that complex structures lost over evolutionary time cannot be regained in the same form [58]. We previously hypothesized that the tooth development programme may initiate in the lower jaw of many living frog species, forming a transient OB and tooth bud rudiments, before being disrupted by a conserved mechanism that originated in proto-frogs [14]. Results presented here, however, suggest that tooth development is fully suppressed in the lower jaw of *O. septentrionalis*. Further analysis of the mechanism underlying this extraordinary evolutionary reversal is constrained by the lack of living specimens and fresh tissues of *G. guentheri*, which is feared extinct [59]. Because *O. septentrionalis* and *G. guentheri* are distant relatives within the frog tree of life (different families, albeit within the same superfamily), a promising avenue of research may be to investigate whether close relatives of *G. guentheri,* especially congeneric species, initiate odontogenic signalling in the lower jaw.

### 3.3. Gene expression patterns overlap between keratinized mouthparts and teeth

All vertebrate skin appendages (i.e. ectodermal or epithelial appendages), including teeth, taste buds, scales, feathers, hair, nails and multiple gland types, are derived from embryonic ectoderm (and sometimes endoderm [60,61]), initiate via epithelial–mesenchymal interactions that form placodes and bud into or out of the mesenchyme [62–64]. Moreover, development of these diverse appendage types deploys the same conserved signalling pathways, including Wnt, Fgf, Bmp, Hh and Eda [64]. These common features suggest that these structures may have evolved from a shared, ancient appendage in stem vertebrates [65,66]. Altering the expression of these signals can modify appendage fate, as in the transition of scales to feathers in birds [67]. Such results demonstrate the lability of this core appendage developmental programme and its capacity for morphological innovation.

The keratinized mouthparts of tadpoles are also ectodermal appendages, although they are rarely recognized as such [68]. The jaw sheaths and keratodonts are derived from embryonic ectoderm, they initiate via formation of oral and aboral epithelial placodes that protrude from condensing mesenchyme [69,70], and our expression data—the first ever reported for keratinized mouthparts in tadpoles—suggest that conserved signalling may regulate their development. For example, *Shh* and *CTNNB1*, which are central to Hh and Wnt signalling, respectively, are often expressed in developing ectodermal appendages [62]. Most vertebrates that undergo metamorphosis, including biphasic teleost fishes, extinct temnospondyl amphibians, salamanders and caecilians, possess true teeth in both larval and adult forms [10,71,72]. Frogs are, therefore, unique in that tadpoles possess a highly specialized feeding apparatus that lack true teeth but is instead composed of keratinized mouthparts. While the origin of the novel tadpole feeding apparatus is unknown, the likely ancestral larval state in crown-group frogs is a tadpole with keratinized mouthparts [27]. If true, two key events occurred during the evolution of proto-anuran larvae: (i) tooth development was delayed from embryogenesis to metamorphosis, and (ii) keratinized mouthparts originated and assumed the larval feeding function of teeth.

The keratinized mouthparts of tadpoles and true teeth of adult frogs were historically regarded as unrelated structures because they differ in cellular anatomy, tissue composition and gross morphology [22]. However, these structures are probably more closely related than previously hypothesized. First, they are both ectodermal appendages and express a similar complement of genes (figures 2–5). Moreover, chimeric grafting experiments conducted nearly a century ago generated salamander larvae with keratinized mouthparts by transplanting frog-embryo ectoderm to the future mouth region of salamander embryos [73–75], demonstrating that these distinct organs can be induced by the same underlying ectomesenchyme signals [76] and therefore share a developmental programme in some capacity. The expression of *Pitx2* in tadpole-keratinized mouthparts (figures 2 and 5) is particularly intriguing because this transcription factor is one of the most crucial regulators of the tooth GRN [5,30,77]. Although *Pitx2* is expressed in a wide range of tissues (e.g. eye, heart, gut and pituitary gland [78]), it is not a key regulator of other ectodermal appendages (scales, hair, feathers, etc. [62,65,79]). This shared expression pattern may indicate instead that the core GRN underlying tooth formation was partially co-opted during the early evolution of frogs and then further modified to form a new appendage type that is exclusive to their larvae: keratinized mouthparts. Co-option occurs during evolution when an ancestral gene or GRN that regulates the development of one structure becomes activated in a new developmental context [80–82] and gives rise to a different, non-homologous structure; it can play a key role in major morphological transitions [83]. Similar expression patterns between a novel structure and a pre-existing organ can be a sign of GRN co-option, but functional experiments are needed to demonstrate that some of the common genes are functionally required for both traits [82]. Knockdown experiments of essential tooth GRN genes during tadpole mouthpart development may be a fruitful place to start.

Keratinized feeding structures have evolved repeatedly among vertebrates, including lampreys and hagfish [84], teleosts [85], frogs and sirenid salamanders [86], and amniotes [48]. In all cases except agnathans, keratinized structures evolved in the oral cavity to functionally replace true teeth that were evolutionarily lost. Expression patterns shared between teeth and tadpole keratinized mouthparts lend additional support to the hypothesis that the origin of keratinized feeding structures in vertebrates is developmentally linked to the tooth GRN in some way [47]. One possibility is that some of the signalling pathways that typically orchestrate odontogenesis are recruited to form keratinized structures but are then unable to participate in tooth development. In birds, Bmp, Shh, Fgf and Wnt signalling is involved in formation of the beak and rhamphotheca [87,88], and some of these signalling pathways may have been diverted from tooth development to rhamphotheca formation [37]. Similarly, FGF4 protein signals are present during development of both tooth buds and baleen plates in the bowhead whale, and the tooth formation signalling cascade may have been co-opted to form baleen during the evolution of mysticete whales [53]. Finally, Hautier *et al.* [89] demonstrated a developmental link between odontogenesis and the formation of keratinized chewing pads in sirenians (manatees and relatives) in which the nerves that typically innervate teeth are re-purposed to innervate the keratinized pad instead. Additional characterization of keratin-to-tooth and tooth-to-keratin transitions among living vertebrates will shed light on the genomic, developmental and anatomical features that may link these highly distinct yet seemingly related tissues.

## 4. Conclusion

Postmetamorphic development of anurans remains an enigma in many respects. The unique juxtaposition of dental conservation in the upper jaw with the complete inhibition of tooth development in the mandible suggests the presence of deeply rooted modules that segregate the jaws. One possible explanation for such decoupling is that yet-to-be identified *cis*-regulatory elements differentially control gene expression patterns associated with odontogenesis in the upper jaw and mandible. Additional work is needed to define this frog-specific evolutionary and developmental anomaly.

## 5. Material and methods

### 5.1. Specimen sampling and histology

All tadpoles were collected from an invasive population in Gainesville, Alachua County, Florida (Florida Fish and Wildlife Conservation Commission permit number LSSC-12-00016C) and reared in captivity at the University of Florida (IACUC protocol no. 202111468). An ontogenetic series across metamorphosis was obtained, ranging from GS 40 to 46 [29]. In total, we investigated the histology of 45 specimens: seven GS 40, nine GS 41, nine GS 42, five GS 43, six GS 44, five GS 45 and four GS 46. Specimens were euthanized using neutral-buffered Tricaine mesylate (MS-222) and fixed in 4% paraformaldehyde at 4°C for 24-48 h. Following fixation, specimens were dehydrated through a diethyl pyrocarbonate phosphate-buffered saline (DEPC PBC)-to-ethanol gradient and then stored in 100% ethanol at −20°C until paraffin sectioning. Specimens were decalcified in 10% ethylenediaminetetraacetic acid (EDTA) for 48 h, embedded in paraffin following standard protocols, and sectioned to 8−10 μm in sagittal or coronal planes using a rotary microtome at the University of Florida (Leica RM2145) or at the University of Dayton (Leica RM2155 or Rankin MCT25). Tissue sections were then either stained using a standard haematoxylin and eosin (H&E) plus Alcian blue protocol (electronic supplementary material, Methods) to evaluate histological anatomy or processed for *in situ* hybridization to characterize gene expression patterns. Following examination of GS 40−46 specimens, we additionally obtained and sectioned multiple embryos to investigate gene expression patterns associated with primary mouth (i.e. stomodeum) formation during GS 20−21.

### 5.2. RNA probe synthesis and section *in situ* hybridization

We designed and synthesized RNA probes for five developmental genes with vital roles in the induction, early differentiation and morphogenesis of teeth in fishes and amniotes [2–4]: *Sox2*, *Pitx2*, *Shh*, *CTNNB1* and *Dlx2*. The OB is marked by expression of *Shh*, *Pitx2* and *Sox2* [2,3,6], and both *CTNNB1* and *Dlx2* are often used as tooth bud markers [4,51]. Digoxigenin-labelled antisense riboprobes were designed using sequence data available from frog genomes published on NCBI. Riboprobes were cloned using *O. septentrionalis* complementary DNA using the following primer sequences: ***Sox2*** (forward GAGACCCATGAACGCCTTCATG; reverse TAGTGCTGGGACATGTGCAG), ***Pitx2*** (forward AGAGACAGAGGAGGCAGAGG; reverse TGCCAGGCTGGAGTTACATG), ***Shh*** (forward GACACCCTAAAAAGCTGACC; reverse CAAATCCAGCCTCCACCGCCAG), ***CTNNB1*** (forward AATTGCTGGTTCGTGCACAC; reverse TCCCATTGCATCTTGCCCAT) and ***Dlx2*** (forward ACCGGAGTGTTTGACAGCTTGG; reverse TGTGATGATGGAGGTGGTGC). Polymerase chain reaction (PCR) clones were ligated into pGEM-T-Easy Vectors (Promega) and used as a template for probe synthesis. Digoxigenin-labelled antisense RNA probes were synthesized through *in vitro* transcription of the PCR templates with T7/SP6 RNA polymerase (Promega) and DIG RNA Labeling Mix (Roche). *In situ* hybridization was performed on the paraffin-embedded tissue sections using a standard protocol (electronic supplementary material, Methods). Specificity of antisense RNA probes was tested by comparing results to those from negative control-sense probes. *In situ* hybridization was performed multiple times for each probe to ensure that expression patterns were reproducible.

## Data Availability

All data are available within the manuscript and in supplementary information. We deposited CT image stacks (TIFF) in MorphoSource (GS 40 tadpole: [90]; GS 42 tadpole: [91]; adult: [92]). Supplementary material is available online [93].

## References

[1] Rücklin M, Donoghue PCJ, Johanson Z, Trinajstic K, Marone F, Stampanoni M. 2012 Development of teeth and jaws in the earliest jawed vertebrates. Nature **491**, 748–751. (10.1038/nature11555) 23075852

[2] Fraser GJ, Graham A, Smith MM. 2004 Conserved deployment of genes during odontogenesis across osteichthyans. Proc. R. Soc. B **271**, 2311–2317. (10.1098/rspb.2004.2878) PMC1691870 15556883

[3] Tucker A, Sharpe P. 2004 The cutting-edge of mammalian development; how the embryo makes teeth. Nat. Rev. Genet. **5**, 499–508. (10.1038/nrg1380) 15211352

[4] Rasch LJ, Martin KJ, Cooper RL, Metscher BD, Underwood CJ, Fraser GJ. 2016 An ancient dental gene set governs development and continuous regeneration of teeth in sharks. Dev. Biol. **415**, 347–370. (10.1016/j.ydbio.2016.01.038) 26845577

[5] Sadier A, Soukup V. 2023 Initiation and periodic patterning of vertebrate dentitions. In Odontodes (ed. D Chen), pp. 215–254. Boca Raton, FL: CRC Press. (10.1201/9781003439653-7)

[6] Juuri E *et al*. 2013 Sox2 marks epithelial competence to generate teeth in mammals and reptiles. Development **140**, 1424–1432. (10.1242/dev.089599) 23462476 PMC3596986

[7] Hovorakova M, Zahradnicek O, Bartos M, Hurnik P, Stransky J, Stembirek J, Tucker AS. 2020 Reawakening of ancestral dental potential as a mechanism to explain dental pathologies. Integr. Comp. Biol. **60**, 619–629. (10.1093/icb/icaa053) 32492167

[8] Soukup V, Tazaki A, Yamazaki Y, Pospisilova A, Epperlein HH, Tanaka EM, Cerny R. 2021 Oral and palatal dentition of axolotl arises from a common tooth-competent zone along the ecto-endodermal boundary. Front. Cell Dev. Biol. **8**, 622308. (10.3389/fcell.2020.622308) 33505974 PMC7829593

[9] Smith MM, Fraser GJ, Mitsiadis TA. 2009 Dental lamina as source of odontogenic stem cells: evolutionary origins and developmental control of tooth generation in gnathostomes. J. Exp. Zool. B **312B**, 260–280. (10.1002/jez.b.21272) 19156674

[10] Paluh DJ *et al*. 2021 Rampant tooth loss across 200 million years of frog evolution. eLife **10**, e66926. (10.7554/elife.66926) 34060471 PMC8169120

[11] Aigler SR, Jandzik D, Hatta K, Uesugi K, Stock DW. 2014 Selection and constraint underlie irreversibility of tooth loss in cypriniform fishes. Proc. Natl Acad. Sci. USA **111**, 7707–7712. (10.1073/pnas.1321171111) 24821783 PMC4040602

[12] Keränen SVE, Kettunen P, Åberg T, Thesleff I, Jernvall J. 1999 Gene expression patterns associated with suppression of odontogenesis in mouse and vole diastema regions. Dev. Genes Evol. **209**, 495–506. (10.1007/s004270050282) 10415326

[13] Wiens JJ. 2011 Re‐evolution of lost mandibular teeth in frogs after more than 200 million years, and re‐evaluating Dollo’s law. Evolution **65**, 1283–1296. (10.1111/j.1558-5646.2011.01221.x) 21521189

[14] Paluh DJ, Dillard WA, Stanley EL, Fraser GJ, Blackburn DC. 2021 Re‐evaluating the morphological evidence for the re‐evolution of lost mandibular teeth in frogs. Evolution **75**, 3203–3213. (10.1111/evo.14379) 34674263 PMC9299036

[15] Davit‐Béal T, Chisaka H, Delgado S, Sire J. 2007 Amphibian teeth: current knowledge, unanswered questions, and some directions for future research. Biol. Rev. **82**, 49–81. (10.1111/j.1469-185x.2006.00003.x) 17313524

[16] Lainoff AJ, Moustakas‐Verho JE, Hu D, Kallonen A, Marcucio RS, Hlusko LJ. 2015 A comparative examination of odontogenic gene expression in both toothed and toothless amniotes. J. Exp. Zool. B **324**, 255–269. (10.1002/jez.b.22594) PMC4401653 25678399

[17] Grieco TM, Hlusko LJ. 2016 Insight from frogs: sonic hedgehog gene expression and a re‐evaluation of the vertebrate odontogenic band. Anat. Rec. **299**, 1099–1109. (10.1002/ar.23378) 27262165

[18] Shaw JP. 1979 The time scale of tooth development and replacement in Xenopus laevis (Daudin). J. Anat. **129**, 323–342. 500489 PMC1233050

[19] Wang S, Stiegler J, Wu P, Chuong CM, Hu D, Balanoff A, Zhou Y, Xu X. 2017 Heterochronic truncation of odontogenesis in theropod dinosaurs provides insight into the macroevolution of avian beaks. Proc. Natl Acad. Sci. USA **114**, 10930–10935. (10.1073/pnas.1708023114) 28973883 PMC5642708

[20] Wang S, Stiegler J, Wu P, Chuong CM. 2020 Tooth vs beak: the evolutionary developmental control of the avian feeding apparatus. In Pennaraptoran theropod dinosaurs: past progress and new frontiers. Bulletin of the American Museum of Natural History, no. 440 (eds M Pittman, X Xu), pp. 205–228. New York: American Museum of Natural History.

[21] Svensson ME, Haas A. 2005 Evolutionary innovation in the vertebrate jaw: a derived morphology in anuran tadpoles and its possible developmental origin. BioEssays **27**, 526–532. (10.1002/bies.20224) 15832380

[22] Luckenbill LM. 1964 Fine structure and development of the horny jaws and teeth of the tadpole (Rana pipiens). PhD thesis, Brown University: Providence, Rhode Island, USA.

[23] Luckenbill LM. 1965 Morphogenesis of the horny jaws of Rana pipiens larvae. Dev. Biol. **11**, 25–49. (10.1016/0012-1606(65)90036-9) 14300093

[24] Kaung HLC. 1975 Development of beaks of Rana pipiens larvae. Anat. Rec. **182**, 401–413. (10.1002/ar.1091820402) 1080023

[25] Kaung HLC, Kollros JJ. 1977 Cell turnover in the beak of Rana pipiens. Anat. Rec. **188**, 361–370. (10.1002/ar.1091880309) 302657

[26] Alibardi L. 2024 Epidermal cell proliferation and differentiation in the beak of tadpoles of Rana dalmatina. Acta Zool. **106**, 205–214. (10.1111/azo.12512)

[27] Altig R. 2006 Discussions of the origin and evolution of the oral apparatus of anuran tadpoles. Acta Herpetol. **1**, 95–105. (10.13128/Acta_Herpetol-1292)

[28] Trueb L. 1966 Morphology and development of the skull in the frog Hyla septentrionalis. Copeia **1966**, 562–573.

[29] Gosner KL. 1960 A simplified table for staging anuran embryos and larvae with notes on identification. Herpetologica **16**, 183–190.

[30] Yu W, Sun Z, Sweat Y, Sweat M, Venugopalan SR, Eliason S, Cao H, Paine ML, Amendt BA. 2020 Pitx2-Sox2-Lef-1 interactions specify progenitor oral/dental epithelial cell signaling centers. Development **147**, v186023. (10.1242/dev.186023) PMC7286298 32439755

[31] Grieco TM. 2013 The developmental basis of variation in tooth and jaw patterning: evolved differences in the Silurana (Xenopus) tropicalis dentition. PhD thesis, UC Berkeley, Berkeley, CA, USA.

[32] Tesche M, Greven H. 2009 Primary teeth in Anura are nonpedicellate and bladed. J. Zool. Syst. Evol. Res. **27**, 1989. (10.1111/j.1439-0469.1989.tb00355.x)

[33] Barlow LA. 1998 The biology of amphibian taste. In Amphibian biology, volume 3: sensory perception (eds H Heatwole, E Dawley), pp. 743–782. Chipping Norton, Australia: Surrey Beatty and Sons.

[34] Bloomquist RF, Parnell NF, Phillips KA, Fowler TE, Yu TY, Sharpe PT, Streelman JT. 2015 Coevolutionary patterning of teeth and taste buds. Proc. Natl Acad. Sci. USA **112**, E5954–62. (10.1073/pnas.1514298112) 26483492 PMC4640805

[35] Dickinson AJG, Sive H. 2006 Development of the primary mouth in Xenopus laevis. Dev. Biol. **295**, 700–713. (10.1016/j.ydbio.2006.03.054) 16678148

[36] Soukup V, Horácek I, Cerny R. 2013 Development and evolution of the vertebrate primary mouth. J. Anat. **222**, 79–99. (10.1111/j.1469-7580.2012.01540.x) 22804777 PMC3552417

[37] Louchart A, Viriot L. 2011 From snout to beak: the loss of teeth in birds. Trends Ecol. Evol. **26**, 663–673. (10.1016/j.tree.2011.09.004) 21978465

[38] Li C, Fraser NC, Rieppel O, Wu XC. 2018 A Triassic stem turtle with an edentulous beak. Nature **560**, 476–479. (10.1038/s41586-018-0419-1) 30135526

[39] Trueb L, Hanken J. 1992 Skeletal development in Xenopus laevis (Anura: Pipidae). J. Morphol. **214**, 1–41. (10.1002/jmor.1052140102) 1433306

[40] Altig R, McDiarmid RW. 1999 Body plan: development and morphology. In Tadpoles: the biology of anuran larvae (eds RW McDiarmid, R Altig), pp. 24–51. Chicago, IL: The University of Chicago Press.

[41] Hertwig O. 1874 Über das Zahnsystem der Amphibien und seine Bedeutung für die Genese des Skelets der Mundhöhle, eine vergleichend anatomische, entwicklungsgeschichtliche Untersuchung. Arch. Für Mikrosk. Anat. Bd **11**, 1–208.

[42] Gillette R. 1955 The dynamics of continuous succession of teeth in the frog (Rana pipiens). Am. J. Anat. **96**, 1–36. (10.1002/aja.1000960102) 14361304

[43] Goin CJ, Hester M. 1961 Studies on the development, succession and replacement of teeth in the frog Hyla cinerea. J. Morphol. **109**, 279–287. (10.1002/jmor.1051090305) 13899582

[44] Fraser GJ, Bloomquist RF, Streelman JT. 2008 A periodic pattern generator for dental diversity. BMC Biol. **6**, 15. (10.1186/1741-7007-6-32) 18625062 PMC2496899

[45] Vonk FJ *et al*. 2008 Evolutionary origin and development of snake fangs. Nature **454**, 630–633. (10.1038/nature07178) 18668106

[46] Sadier A, Sears KE, Womack M. 2021 Unraveling the heritage of lost traits. J. Exp. Zool. B **338**, 107–118. (10.1002/jezb.23030) 33528870

[47] Lewis ZR, Kerney R, Hanken J. 2022 Developmental basis of evolutionary lung loss in plethodontid salamanders. Sci. Adv. **8**, eabo6108. (10.1126/sciadv.abo6108) 35977024 PMC9385146

[48] Davit‐Béal T, Tucker AS, Sire J. 2009 Loss of teeth and enamel in tetrapods: fossil record, genetic data and morphological adaptations. J. Anat. **214**, 477–501. (10.1111/j.1469-7580.2009.01060.x) 19422426 PMC2736120

[49] Chen Y *et al*. 2000 Conservation of early odontogenic signaling pathways in Aves. Proc. Natl Acad. Sci. USA **97**, 10044–10049. (10.1073/pnas.160245097) 10954731 PMC27667

[50] Tokita M, Chaeychomsri W, Siruntawineti J. 2013 Developmental basis of toothlessness in turtles: insight into convergent evolution of vertebrate morphology. Evolution **67**, 260–273. (10.1111/j.1558-5646.2012.01752.x) 23289576

[51] Peterková R, Peterka M, Viriot L, Lesot H. 2002 Development of the vestigial tooth primordia as part of mouse odontogenesis. Connect. Tissue Res. **43**, 120–128. (10.1080/03008200290000745) 12489147

[52] Stock DW, Jackman WR, Trapani J. 2006 Developmental genetic mechanisms of evolutionary tooth loss in cypriniform fishes. Development **133**, 3127–3137. (10.1242/dev.02459) 16831836

[53] Jackman WR, Miranda Portillo LS, Cox CK, Ambrosio A, Gibert Y. 2024 Blocking endogenous retinoic acid degradation induces oral tooth formation in zebrafish. Proc. Natl Acad. Sci. USA **121**, e2321162121. (10.1073/pnas.2321162121) 38446853 PMC10945834

[54] Thewissen JGM, Hieronymus TL, George JC, Suydam R, Stimmelmayr R, McBurney D. 2017 Evolutionary aspects of the development of teeth and baleen in the bowhead whale. J. Anat. **230**, 549–566. (10.1111/joa.12579) 28070906 PMC5345624

[55] Dickinson AJG, Sive HL. 2009 The Wnt antagonists Frzb-1 and Crescent locally regulate basement membrane dissolution in the developing primary mouth. Development **136**, 1071–1081. (10.1242/dev.032912) 19224982 PMC2685928

[56] Tabler JM, Bolger TG, Wallingford J, Liu KJ. 2014 Hedgehog activity controls opening of the primary mouth. Dev. Biol. **396**, 1–7. (10.1016/j.ydbio.2014.09.029) 25300580 PMC4252736

[57] AmphibiaWeb. 2025 AmphibiaWeb. Berkeley, CA: University of California. See https://amphibiaweb.org (accessed 15 January 2025).

[58] Gould SJ. 1970 Dollo on Dollo’s law: irreversibility and the status of evolutionary laws. J. Hist. Biol. 189–212. (10.1007/BF00137351) 11609651

[59] Riva I, Lansac C, Cepeda B, Cantillo G, Luca J, González L, Márquez R, Burrowes PA. 2020 Forensic bioacoustics? The advertisement calls of two locally extinct frogs from Colombia. Amphib. Reptile Conserv. **14**, 177–188.

[60] Soukup V, Epperlein HH, Horácek I, Cerny R. 2008 Dual epithelial origin of vertebrate oral teeth. Nature **455**, 795–798. (10.1038/nature07304) 18794902

[61] Barlow LA. 2025 Development of ectodermal and endodermal taste buds. Dev. Biol. **518**, 20–27. (10.1016/j.ydbio.2024.10.005) 39486632 PMC11703678

[62] Pispa J, Thesleff I. 2003 Mechanisms of ectodermal organogenesis. Dev. Biol. **262**, 195–205. (10.1016/s0012-1606(03)00325-7) 14550785

[63] Fraser GJ, Cerny R, Soukup V, Bronner‐Fraser M, Streelman JT. 2010 The odontode explosion: the origin of tooth‐like structures in vertebrates. BioEssays **32**, 808–817. (10.1002/bies.200900151) 20730948 PMC3034446

[64] Biggs LC, Mikkola ML. 2014 Early inductive events in ectodermal appendage morphogenesis. Semin. Cell Dev. Biol. **25–26**, 11–21. (10.1016/j.semcdb.2014.01.007) 24487243

[65] Dhouailly D, Godefroit P, Martin T, Nonchev S, Caraguel F, Oftedal O. 2019 Getting to the root of scales, feather and hair: as deep as odontodes? Exp. Dermatol. **28**, 503–508. (10.1111/exd.13391) 28603898

[66] Aman AJ, Fulbright AN, Parichy DM. 2018 Wnt/β-catenin regulates an ancient signaling network during zebrafish scale development. eLife **7**, e37001. (10.7554/elife.37001) 30014845 PMC6072442

[67] Cooper RL, Milinkovitch MC. 2023 Transient agonism of the sonic hedgehog pathway triggers a permanent transition of skin appendage fate in the chicken embryo. Sci. Adv. **9**, eadg9619. (10.1126/sciadv.adg9619) 37196093 PMC10191425

[68] Holthaus KB, Steinbinder J, Sachslehner AP, Eckhart L. 2025 Skin appendage proteins of tetrapods: building blocks of claws, feathers, hair and other cornified epithelial structures. Animals **15**, 457. (10.3390/ani15030457) 39943227 PMC11816140

[69] Thibaudeau DG, Altig R. 1988 Sequence of ontogenetic development and atrophy of the oral apparatus of six anuran tadpoles. J. Morphol. **197**, 63–69. 29890793 10.1002/jmor.1051970106

[70] Tachibana T. 1979 The Merkel cell in the labial ridge epidermis of the anuran tadpole. Arch. Histol. Jpn **42**, 129–140. (10.1679/aohc1950.42.129) 464748

[71] Dunn JR. 1984 The utility of developmental osteology in taxonomic and systematic studies of teleost larvae: a review. Natl Oceanic Atmos. Adm. Tech. Rep. Natl. Mar. Fish. Serv. Circ. **450**, 1–19.

[72] Schoch RR, Witzmann F. 2024 The evolution of larvae in temnospondyls and the stepwise origin of amphibian metamorphosis. Biol. Rev. **99**, 1613–1637. (10.1111/brv.13084) 38599802

[73] Spemann H, Schotte O. 1932 Über xenoplastische Transplantation als Mittel zur Analyse der embryonalen Induktion. Die Naturwissenschaften **20**, 463–467.

[74] Rotmann E. 1935 Der Anteil von Induktion und reagierended Gewede an der Entwicklung des Haftfadens. W Roux Arch. Entwicklungsmechanic **133**, 193–124. 10.1007/BF00593030 28354979

[75] Henzen W. 1957 Transplantationen zur entwicklungsphysiologischen Analyse der larvalen Mundorgane bei Bombinator und Triton. Wilhelm Roux’ Arch. Für Entwicklungsmechanik Der Org. **149**, 387–442. 10.1007/BF00573725 28354634

[76] Hall BK. 1984 Developmental mechanisms underlying the formation of atavisms. Biol. Rev. **59**, 89–122. (10.1111/j.1469-185x.1984.tb00402.x) 6367843

[77] Ye Q, Bhojwani A, Hu JK. 2022 Understanding the development of oral epithelial organs through single cell transcriptomic analysis. Development **149**, v200539. (10.1242/dev.200539) PMC9481975 35831953

[78] Gage PJ, Suh H, Camper SA. 1999 Dosage requirement of Pitx2 for development of multiple organs. Development **126**, 4643–4651. (10.1242/dev.126.20.4643) 10498698

[79] Duverger O, Morasso MI. 2008 Role of homeobox genes in the patterning, specification, and differentiation of ectodermal appendages in mammals. J. Cell. Physiol. **216**, 337–346. (10.1002/jcp.21491) 18459147 PMC2561923

[80] True JR, Carroll SB. 2002 Gene co-option in physiological and morphological evolution. Annu. Rev. Cell Dev. Biol. **18**, 53–80. (10.1146/annurev.cellbio.18.020402.140619) 12142278

[81] McQueen E, Rebeiz M. 2020 On the specificity of gene regulatory networks: how does network co-option affect subsequent evolution? Curr. Topics Dev. Biol. Gene Regul. Netw. **139**, 375–405. (10.1016/bs.ctdb.2020.03.002) PMC8382235 32450967

[82] DiFrisco J, Wagner GP, Love AC. 2023 Reframing research on evolutionary novelty and co-option: character identity mechanisms versus deep homology. Semin. Cell Dev. Biol. **145**, 3–12. (10.1016/j.semcdb.2022.03.030) 35400563

[83] McKenna KZ, Wagner GP, Cooper KL. 2021 A developmental perspective of homology and evolutionary novelty. Curr. Topics Dev. Biol. **141**, 1–38. (10.1016/bs.ctdb.2020.12.001) 33602485

[84] Grohganz M. 2024 The evolutionary origin of teeth. PhD thesis, University of Bristol, Bristol, UK.

[85] Roberts TR. 1982 Unculi (horny projections arising from single cells), an adaptive feature of the epidermis of ostariophysan fishes. Zool. Scr. **11**, 55–476.

[86] Schwarz D, Fedler MT, Lukas P, Kupfer A. 2021 Form and function of the feeding apparatus of sirenid salamanders (Caudata: Sirenidae): three-dimensional chewing and herbivory? Zool. Anz. **295**, 99–116. (10.1016/j.jcz.2021.09.008)

[87] Wu P, Jiang TX, Suksaweang S, Widelitz RB, Chuong CM. 2004 Molecular shaping of the beak. Science **305**, 1465–1466. (10.1126/science.1098109) 15353803 PMC4380220

[88] Schneider RA. 2024 Cellular, molecular, and genetic mechanisms of avian beak development and evolution. Annu. Rev. Genet. **58**, 433–454. (10.1146/annurev-genet-111523-101929) 39227135 PMC11777486

[89] Hautier L, Gomes Rodrigues H, Ferreira-Cardoso S, Emerling CA, Porcher ML, Asher RJ, Portela Miguez R, Delsuc F. 2023 From teeth to pad: tooth loss and development of keratinous structures in sirenians. Proc. R. Soc. B **290**, 20231932. (10.1098/rspb.2023.1932) PMC10685118 38018114

[90] MorphoSource. 2025 Media 000706057: Gosner stage 40 tadpole. https://www.morphosource.org/concern/media/000706057?locale=en

[91] MorphoSource. 2025 Media 000706053: Gosner stage 42 tadpole. https://www.morphosource.org/concern/media/000706053?locale=en

[92] Blackburn DC *et al*. 2024 Increasing the impact of vertebrate scientific collections through 3D-imaging: the openvertebrate (oVert) thematic collections network. Bioscience **74**, 169–186. (10.17602/M2/M34831) 38560620 PMC10977868

[93] Paluh DJ, Brinkman M, Gilliam-Beale K, Salcedo-Recio D, Szafranski J, Hanken J *et al*. 2025 Supplementary material from: The metamorphic transition of the frog mouth: from tadpole keratinized mouthparts to adult teeth. Figshare. (10.6084/m9.figshare.c.7979938) PMC12404811 40904991

